# A novel tetracycline-responsive transgenic mouse strain for skeletal muscle-specific gene expression

**DOI:** 10.1186/s13395-018-0181-y

**Published:** 2018-10-27

**Authors:** Masahiro Iwata, Davis A. Englund, Yuan Wen, Cory M. Dungan, Kevin A. Murach, Ivan J. Vechetti, Christopher B. Mobley, Charlotte A. Peterson, John J. McCarthy

**Affiliations:** 1 The Center for Muscle Biology, University of Kentucky, Lexington, KY 40536 USA; 2 Department of Rehabilitation Sciences, College of Health Sciences, University of Kentucky, Lexington, KY 40536 USA; 3 Department of Physiology, College of Medicine, University of Kentucky, 800 Rose Street, Medical Science Building, Rm: MS-607A, Lexington, KY 40536 USA; 4 Department of Rehabilitation, Faculty of Health Sciences, Nihon Fukushi University, 26-2 Higashihaemi-cho, Handa, 475-0012 Japan

**Keywords:** Skeletal muscle-specific, Tetracycline-responsive

## Abstract

**Background:**

The tetracycline-responsive system (Tet-ON/OFF) has proven to be a valuable tool for manipulating gene expression in an inducible, temporal, and tissue-specific manner. The purpose of this study was to create and characterize a new transgenic mouse strain utilizing the human skeletal muscle α-actin (HSA) promoter to drive skeletal muscle-specific expression of the reverse tetracycline transactivator (rtTA) gene which we have designated as the HSA-rtTA mouse.

**Methods:**

To confirm the HSA-rtTA mouse was capable of driving skeletal muscle-specific expression, we crossed the HSA-rtTA mouse with the tetracycline-responsive histone H2B-green fluorescent protein (H2B-GFP) transgenic mouse in order to label myonuclei.

**Results:**

Reverse transcription-PCR confirmed skeletal muscle-specific expression of rtTA mRNA, while single-fiber analysis showed highly effective GFP labeling of myonuclei in both fast- and slow-twitch skeletal muscles. Pax7 immunohistochemistry of skeletal muscle cross-sections revealed no appreciable GFP expression in satellite cells.

**Conclusions:**

The HSA-rtTA transgenic mouse allows for robust, specific, and inducible gene expression across muscles of different fiber types. The HSA-rtTA mouse provides a powerful tool to manipulate gene expression in skeletal muscle.

## Background

Since the original description, the tetracycline-responsive system (Tet-ON/OFF) has proven to be a powerful tool in biomedical research because of the ability to manipulate gene expression within the mouse in both a temporal and tissue-specific manner [1, 2]. Although a number of skeletal muscle-specific Tet-ON/OFF mice have been described, they have used promoters that drive primarily fast-twitch, type II gene expression; in addition, these mice are not readily available [3, 4]. To address these limitations, we generated a transgenic mouse which uses the human skeletal muscle α-actin (HSA) promoter to drive skeletal muscle-specific expression of the reverse-tetracycline transactivator (rtTA) which we have designated as the HSA-rtTA mouse. To validate the HSA-rtTA mouse, we crossed it with the tetracycline-responsive histone H2B-green fluorescent protein (TRE-H2B-GFP) mouse to easily visualize and quantify myonuclear GFP expression following doxycycline treatment [5]. As expected, rtTA mRNA was highly expressed in skeletal muscle as > 95% of myonuclei were GFP-positive in both type I and type II muscles. Importantly, an extremely small number of satellite cells appeared to be GFP-positive in soleus muscle cross-section, thus confirming the ability of the HSA-rtTA mouse to drive robust skeletal muscle-specific expression of a tetracycline-responsive gene of interest.

## Methods

### Generating the HSA-rtTA transgenic mouse

**Fig. 1:**
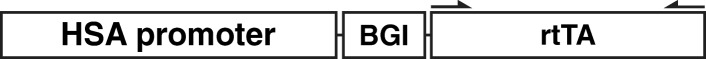
A schematic of the HSA-rtTA transgene. The promoter and first exon (− 2,000 to + 239 relative to the transcription start site) of the human skeletal muscle α-actin (HSA) gene regulates expression of an optimized reverse tetracycline transactivator (rtTA) gene which has been reported to be sevenfold more active and 100-fold more doxycycline sensitive than the original Tet-On system [8]. The β-globin intron ΙΙ (BGI) and poly(A) tail were incorporated into the transgene to ensure proper splicing and transcript stability, respectively. The positions of the PCR primers used for genotyping are indicated by half-arrows

As previously described by us for the HSA-MerCreMer transgene, the promoter and first exon (− 2,000 to + 239 relative to the transcription start site) of the human skeletal muscle α-actin (HSA) gene was amplified from human genomic DNA (Promega, Madison, WI, USA) and cloned into the *Cla*I site of the SG5 expression vector (Agilent Technologies, Santa Clara, CA, USA) upstream of the β-globin intron II [6]. The rtTA cDNA was amplified from the pCMV-Tet3G expression vector (Takara Bio, Mountain View, CA, USA) and cloned into the EcoRI/BamHI sites of the pSG5-HSA plasmid to generate the pSG5-HSA-rtTA; the rtTA insert was subsequently sequenced for verification. The HSA-rtTA transgene (Fig. 1) was released from the plasmid by *Hin*dIII/*Nsi*I enzyme digestion, gel-purified using the QIAquick Gel Extraction Kit according to the manufacturer’s directions (Qiagen, Valencia, CA, USA), and then provided to the University of Michigan Transgenic Animal Model Core for microinjection. F1 generation pups were screened by PCR for the presence of the rtTA sequence using genomic DNA isolated from tail snips with the following primers: F, 5′ATGTCTAGACTGGACAAG AGCA AAG-3′; R, 5′-TTACCCGGGGAGCATGTC-3′ generating a product of 747 bp. Eight F1 pups were positive for the HSA-rtTA transgene and subsequently crossed to the TRE-H2B-GFP mouse (The Jackson Laboratory, stock number 005104) to determine the ability to drive H2B-GFP expression following doxycycline treatment. Of the eight founder lines, line 6 was identified as driving robust H2B-GFP expression in both slow- and fast-twitch muscles of the lower hind limbs and was further characterized as described below. For convenience, the HSA-rtTA/TRE-H2B-GFP mouse is referred to as the HSA-GFP mouse.

### Doxycycline treatment

To induce H2B-GFP expression, 3–10-month-old HSA-GFP mice were administered doxycycline (0.5 mg/mL) in drinking water supplemented with 2% sucrose for 3 weeks. Tissue was collected immediately upon completion of doxycycline treatment. To determine the earliest time of GFP induction, skeletal muscle was collected after 12 h or 24 h following doxycycline administration.

### Analysis of rtTA gene expression

Total RNA was isolated from skeletal muscles (gastrocnemius, plantaris, soleus, extensor digitorum longus (EDL), tibialis anterior (TA), diaphragm and heart, and non-muscle tissue (brain, liver, lung, stomach, spleen, kidney, and fat) of HSA-GFP mice. Tissue was immediately frozen in liquid nitrogen upon excision and subsequently homogenized using a Bullet Blender (Next Advance Inc., Averill Park, NY, USA) in Direct-zol (Zymo Research, Irvine, CA, USA) according to the manufacturer’s instructions. Total RNA concentration and quality were determined by nanoVue spectrophotometer (GE Healthcare, USA). cDNA was synthesized from 1 μg of total RNA using the SuperScript® VILO IV™ (ThermoFisher Scientific, Waltham, MA, USA) according to the manufacturer’s instructions. PCR analysis of rtTA mRNA accumulation used the following primers: F, 5′- GAGGAACAGGAGC ATCAAGTAG-3′; R, 5′- GTCAGCAGGCAGCATATCA-3′ and generated a 270 bp product.

### Single fiber analysis

GFP+ and GFP− myonuclei were counted on isolated single muscle fibers as previously described by us [7]. Briefly, hind limb muscles were fixed in situ at resting length in 4% paraformaldehyde for 48 h. Fixed whole muscles were removed from the hind limb, dissected, and dissociated in 40% sodium hydroxide with manual manipulation at room temperature. Isolated fibers were then stained with DAPI and carefully pipetted on to glass slides and covered using Vectashield (Vector Laboratories, Burlingame, CA, USA).

### Immunohistochemistry

For immunohistochemistry (IHC) analyses, the various hind limb muscles were covered in Tissue-Tek optimal cutting temperature compound (Sakura Finetek, Torrance, CA, USA) and pinned at resting length to a cork covered in aluminum foil. Muscles were frozen in liquid nitrogen-cooled isopentane and stored at − 80 °C. Muscles were sectioned at the mid-belly on a cryostat at − 23 °C. Frozen muscle sections (7 μm) were air-dried for at least 1 h and stored at − 20 °C. For Pax7/DAPI IHC, muscles were first fixed in 4% paraformaldehyde for 7 min and then subjected to epitope retrieval. Following epitope retrieval in sodium citrate (10 mM, pH 6.5) for 20 min at 92 °C, endogenous peroxidases were blocked for 7 min with 3% hydrogen peroxide in phosphate-buffered saline (PBS), followed by 1 h with 1% Tyramide Signal Amplification (TSA) blocking reagent (TSA kit, T20935, Invitrogen) supplemented with Mouse-on-Mouse (MoM) IgG blocking reagent (Vector Laboratories, Burlingame, CA, USA). Sections were washed in PBS and incubated overnight with mouse anti-Pax7 IgG1 antibody (1:100, Developmental Studies Hybridoma Bank (DSHB), Iowa City, IA, USA) and chicken anti-GFP antibody (1:200, Abcam, Cambridge, MA, USA) diluted in 1% TSA blocking reagent. It was necessary to use anti-GFP antibody to detect GFP expression because the antigen retrieval process quenched the GFP signal. The following day, sections were washed with PBS, incubated for 70 min in goat anti-mouse IgG1 biotinylated secondary antibody (1:1000, 115-065-205, Jackson ImmunoResearch, West Grove, PA, USA) and anti-chicken GFP secondary antibody, (1:250, Abcam), washed in PBS, incubated for 1 h in streptavidin-horseradish peroxidase (1:500, S-911, Invitrogen) diluted in PBS, washed again in PBS, then incubated for 15 min in TSA Alexa Fluor 594 (1:500, TSA kit, Invitrogen) in the supplied amplification diluents. Sections were stained with DAPI (1:10,000 in PBS, D35471, Invitrogen) for 5 min and mounted with VectaShield fluorescent mounting media.

### Image acquisition and quantification

GFP+/DAPI+ and GFP−/DAPI+ myonuclei from ~ 10 isolated fibers from four doxycycline-treated mice (two male and two female) were counted for each muscle, resulting in a range of 202–452 myonuclei being analyzed across muscles. Twelve to 15 single fibers from two untreated mice (1 male and 1 female) were analyzed for each muscle, resulting in a range of 254–600 myonuclei being analyzed across muscles. For the time course analysis, 35 diaphragm fibers (*n* = 2) were counted representing a total of 906 myonuclei, whereas 37 plantaris fibers (*n* = 2) were counted representing a total of 1294 myonuclei. For IHC, images were captured at × 20 magnification using a Zeiss upright fluorescent microscope (Zeiss Axio Imager M1, Oberkochen, Germany). Whole muscle sections were obtained using the mosaic function in Zeiss Zen 2.3 imaging software. Satellite cells (Pax7+/DAPI+) and GFP+ satellite cells were identified manually using Zen software tools.

## Results

### Skeletal muscle-specific rtTA transgene

To generate the skeletal muscle-specific rtTA transgene for microinjection, we cloned downstream of the human skeletal muscle α-actin (HSA) promoter a third generation rtTA gene that was reported to be sevenfold more active and 100-fold more doxycycline sensitive than the original rtTA [8]. A schematic of the HSA-rtTA transgene is shown in Fig. 1.

### Skeletal muscle-specific expression of rtTA mRNA

**Fig. 2:**
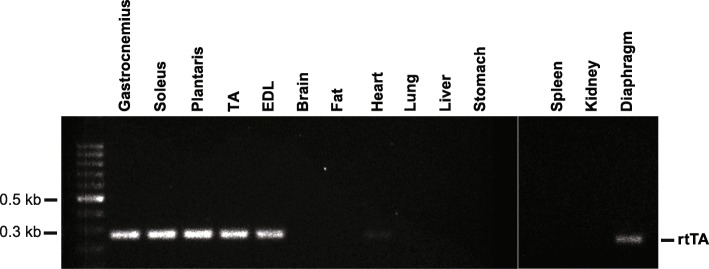
Skeletal muscle-specific expression of rtTA. PCR analysis of rtTA mRNA expression of different tissues from the HSA-GFP transgenic mouse showed high levels of expression in skeletal muscle (gastrocnemius, soleus, plantaris, tibialis anterior (TA), and extensor digitorum longus (EDL)), modest expression in the diaphragm, very low expression in the heart, and not detectable in non-muscle tissue (brain, fat, lung, liver, stomach, spleen, and kidney)

We determined by reverse transcription-PCR the expression of rtTA mRNA in several hind limb muscles, the diaphragm, fat, and several other non-muscle organs. As shown in Fig. 2, rtTA mRNA was highly expressed in all of the hind limb muscles examined and to a lesser extent in the diaphragm and heart. As expected, rtTA expression was not detectable in any non-muscle tissues.

### Effective labeling of myonuclei in hind limb skeletal muscles

**Fig. 3:**
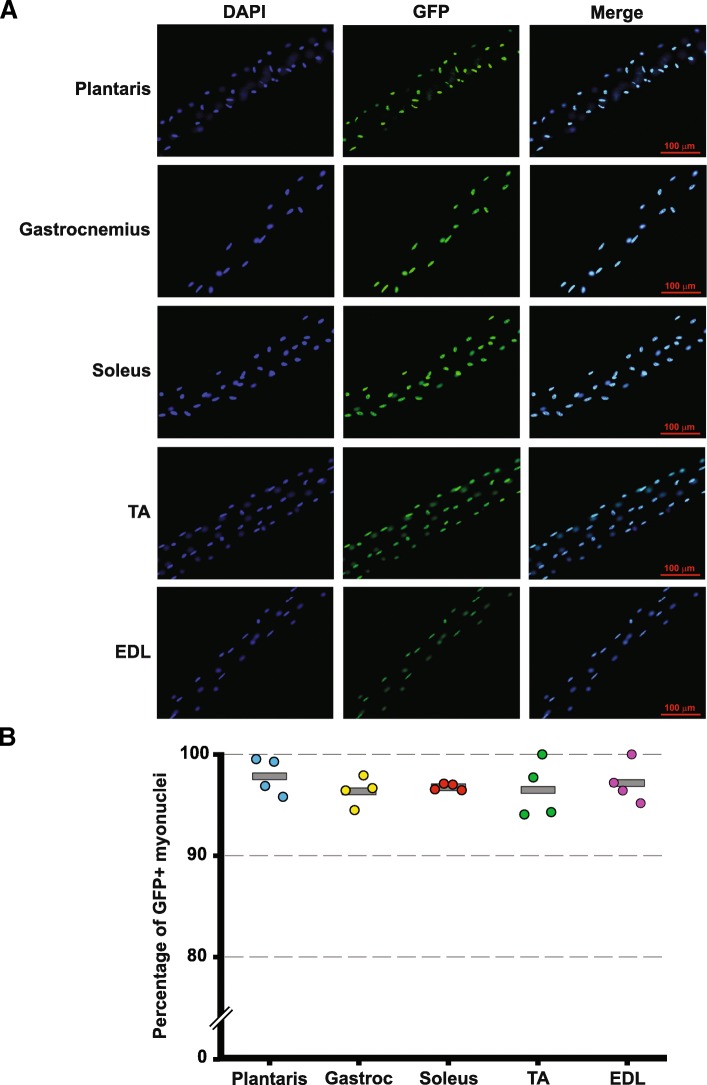
HSA-rtTA transgene drives robust myofiber expression of tetracycline-responsive H2B-GFP transgene. **a** Representative single fiber images of hind limb muscles taken from HSA-GFP mice (*n* = 4) treated with doxycycline. Single fiber images show robust myonuclear GFP expression in muscles composed of slow- and fast-twitch fibers. **b** Quantification of GFP+ myonuclei of single fibers from hind limb skeletal muscles (plantaris, gastrocnemius, soleus, tibialis anterior (TA), and extensor digitorum longus (EDL)) of HSA-GFP mice showed greater than 95% of all DAPI+ myonuclei within a fiber were GFP+. The gray bar represents the average percentage of GFP-positive myonuclei (*n* = 4) for each muscle

Having established that rtTA was highly enriched in skeletal muscle, we sought to determine how effective the HSA-rtTA transgene was in driving H2B-GFP expression in response to doxycycline treatment. Given that H2B-GFP is nuclearly localized, we used the percentage of myonuclei that were GFP+ on single fibers as a measure of the effectiveness of the HSA-rtTA transgene to induce expression of a tetracycline-responsive gene. Following fixation, single fibers were isolated from the plantaris, gastrocnemius, soleus, tibialis anterior (TA), and extensor digitorum longus (EDL) of doxycycline-treated mice and then stained with DAPI to identify myonuclei. As shown in Fig. 3a, b, greater than 95% (range of 96.4–97.9%) of myonuclei were GFP+ across all muscles from doxycycline-treated HSA-GFP mice. We observed no GFP+ myonuclei in skeletal muscle single fibers of untreated HSA-GFP mice demonstrating tight regulation of tetracycline-responsive H2B-GFP gene (data not shown). These findings confirm the HSA-rtTA mouse is capable of driving robust expression of a tetracycline-responsive gene in adult skeletal muscles composed of both slow- and fast-twitch fibers.

### GFP labeling is highly specific to myonuclei

**Fig. 4:**
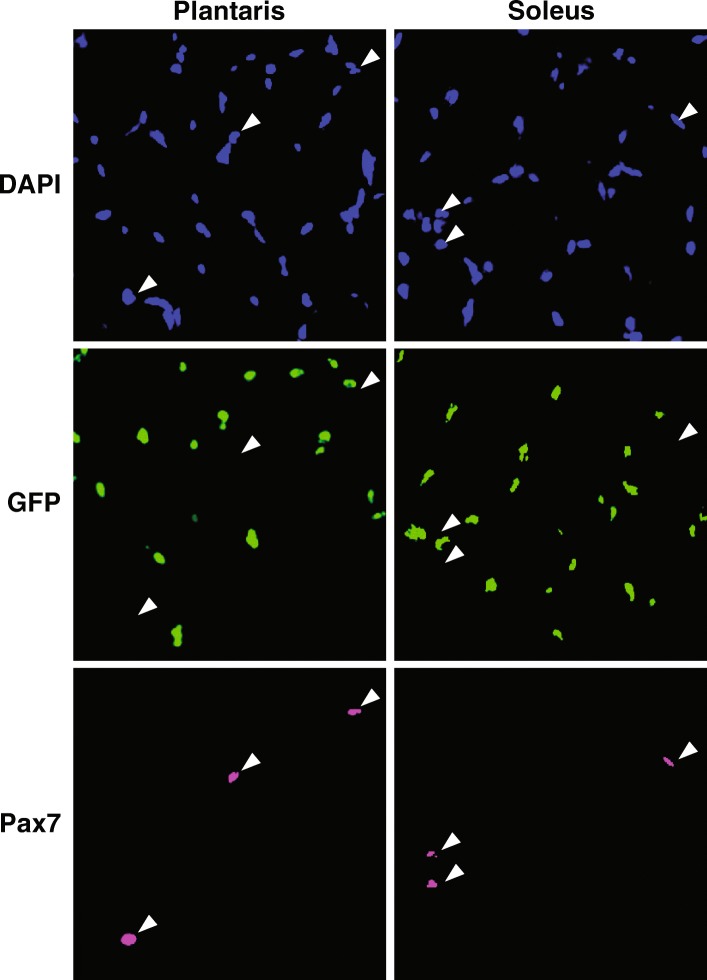
GFP expression is specific to myonuclei in HSA-GFP mice. Representative muscle cross-section images of the plantaris and soleus muscles from HSA-GFP mice treated with doxycycline (*n* = 3). As indicated by white arrows, DAPI+/GFP+ myonuclei (green) did not show co-localization with DAPI+/Pax7+ satellite cells (pink). These results confirm that the HSA-rtTA transgene is able to drive myofiber-specific expression of a tetracycline-response gene

To determine if the HSA-rtTA drove expression of the H2B-GFP transgene in satellite cells, we performed immunohistochemistry on both soleus and plantaris muscle cross-sections for DAPI, Pax7, and GFP. As shown in Fig. 4, GFP labeling did not localize with Pax7 staining; however, in the soleus, of the 190 satellite cells counted, three Pax7+ cells appeared to be GFP+. These results demonstrate the HSA-rtTA drives highly myofiber-specific expression of a tetracycline-responsive transgene.

### Rapid GFP labeling of myonuclei

**Fig. 5:**
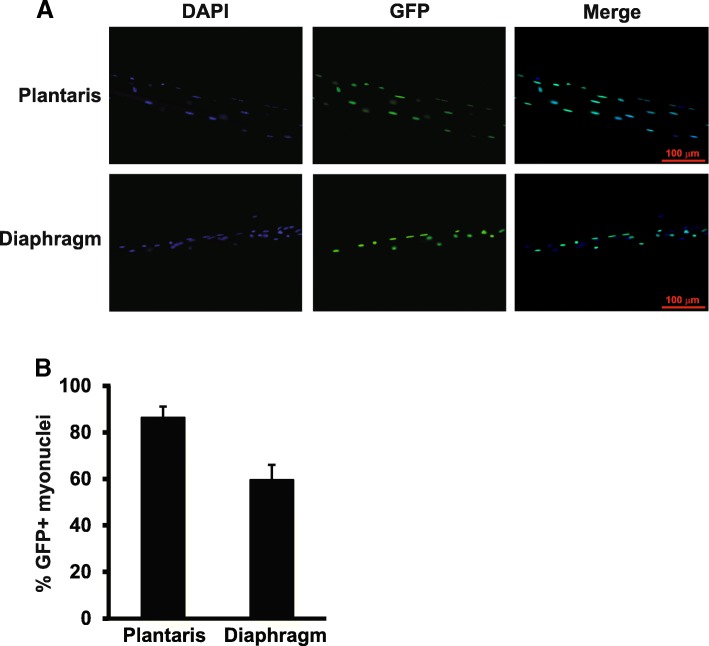
Rapid GFP labeling of myonuclei. **a** Representative single fiber images of plantaris and diaphragm muscles taken from HSA-GFP mice (*n* = 2) treated with doxycycline for 24 h. **b** Quantification of GFP+ myonuclei showed ~ 90% of myonuclei were GFP-positive in myofibers from the plantaris with 60% of myonuclei GFP-positive in myofibers isolated from the diaphragm

To determine the time course of GFP labeling of myonuclei, skeletal muscle was collected from HAS-GFP mice after 12 or 24 h of doxycycline treatment. As shown in Fig. 5a, b, approximately 90% of myonuclei in the plantaris muscle were GFP-positive after 24 h of doxycycline treatment; in contrast, GFP expression was not detected following 12 h of doxycycline administration (data not shown). We also examined whether GFP expression followed the same time course of induction given the modest expression of rtTA mRNA in the diaphragm (see Fig. 2). While GFP expression was not as robust as that observed in the plantaris, 60% of myonuclei of the diaphragm were GFP-positive following 24 h of doxycycline treatment, consistent with the lower rtTA mRNA expression (see Fig. 5a–b).

## Discussion

The purpose of this study was to characterize a new skeletal muscle-specific Tet-ON mouse. The human skeletal muscle α-actin (HSA) promoter was used to drive skeletal muscle-specific expression of the reverse-tetracycline transactivator (rtTA), designated as the HSA-rtTA mouse. The HSA promoter contains 2,000 bp of human skeletal α-actin 5′-flanking sequence plus the first exon and 149 bp of the first intron and was first reported by Muscat and Kedes to promote robust, skeletal muscle-specific expression [9]. We choose to use the HSA promoter because we previously showed it was able to drive effective Cre-mediated recombination in both slow- and fast-twitch fibers with minimal expression in the heart [6]. This is an important improvement over a previous skeletal muscle-specific Tet-ON mouse (MCK-rtTA) which only allowed over-expression of a gene of interest in fast-twitch, type IIb fibers [10]. Together with the HSA-CreER mouse, the HSA-rtTA mouse now provides the ability to perform loss- and gain-of-function studies, respectively, to determine the in vivo function of a gene of interest in skeletal muscle fibers [6, 11]. The complement to these two inducible, skeletal muscle-specific mice are the satellite cell-specific inducible Cre and Tet-ON mice; however, while the satellite cell-specific Cre mouse has been extensively used, to the best of our knowledge, the satellite cell-specific Tet-ON mouse has yet to be fully characterized [12–15]. Collectively, these inducible, skeletal muscle-, and satellite cell-specific mice provide powerful tools to manipulate in vivo gene expression to identify and better understand the mechanisms regulating skeletal muscle biology in health and disease.

While 3 weeks of doxycycline treatment was able to induce > 95% GFP labeling of myonuclei, we wanted to know the earliest time point when GFP expression could be detected following doxycycline administration. As shown in Fig. 5, single fiber analysis revealed myonuclear GFP expression was observed as early as 24 h post-doxycycline exposure; in contrast, we observed no GFP expression at 12 h post-doxycycline treatment (data not shown). These results demonstrate the HSA-rtTA mouse is very responsive to doxycycline and will provide the ability to study the relative early (~ 24 h) effects of gene activation on a given biological process. For example, the HSA-rtTA mouse could be used to study how the early (~ 24 h) activation of Akt1 (using the tetracycline-responsive, constitutively active Akt1 mouse, TRE-myrAkt1) affects the hypertrophic response in skeletal muscle as Akt1 is typically not activated until 48 h [16, 17].

In contrast to the strong rtTA expression in hind limb muscles, there was modest, but detectable, expression of rtTA mRNA in the diaphragm. We do not know the reason why rtTA expression is lower in the diaphragm compared to hind limb muscles, but it may reflect a limitation of the HSA promoter to drive high levels of expression in the diaphragm. We also found comparatively lower expression of Cre in the diaphragm of the HSA-CreER mouse, consistent with the idea that the HSA promoter is not as robust in the diaphragm as it is in other muscles. Despite low rtTA expression, we still observed ~ 60% GFP labeling of diaphragm myonuclei after only 24 h of doxycycline treatment. This result indicates the HSA-rtTA mouse is a useful tool for investigators studying the diaphragm.

The HSA-rtTA transgenic mouse allows for inducible, myofiber-specific gene expression in both slow- and fast-twitch muscles. The HSA-rtTA mouse will provide researchers with a powerful tool to reversibly induce gene expression in an effort to better understand skeletal muscle biology. The HSA-rtTA mouse will be freely available upon request.

## Abbreviations

dox: Doxycycline
EDL: Extensor digitorum longus
GFP: Green fluorescent protein
H2B: Histone H2B
HSA: Human skeletal muscle α-actin
IHC: Immunohistochemistry
rtTA: Reverse tetracycline transactivator
TA: Tibialis anterior
Tet-ON/OFF: Tetracycline-responsive system
